# The role of complement in tumor immune tolerance and drug resistance: a double-edged sword

**DOI:** 10.3389/fimmu.2025.1529184

**Published:** 2025-01-31

**Authors:** Ronghui Yang, Di Fu, Aijun Liao

**Affiliations:** ^1^ Department of Blood Transfusion, The First Hospital of China Medical University, Shenyang, Liaoning, China; ^2^ Department of Hematology, Shengjing Hospital of China Medical University, Shenyang, Liaoning, China; ^3^ Department of General Practice, Central Hospital Affiliated to Shenyang Medical College, Shenyang, Liaoning, China

**Keywords:** complement system, tumor, anti-tumor, immune tolerance, drug resistance

## Abstract

The domain of cancer treatment has persistently been confronted with significant challenges, including those associated with recurrence and drug resistance. The complement system, which serves as the foundation of the innate immune system, exhibits intricate and nuanced dual characteristics in the evolution of tumors. On the one hand, the complement system has the capacity to directly inhibit cancer cell proliferation via specific pathways, thereby exerting a beneficial anti-tumor effect. Conversely, the complement system can also facilitate the establishment of an immune escape barrier for cancer cells through non-complement-mediated mechanisms, thereby protecting them from eradication. Concurrently, the complement system can also be implicated in the emergence of drug resistance through a multitude of complex mechanisms, directly or indirectly reducing the efficacy of therapeutic interventions and facilitating the progression of cancer. This paper analyses the role of the complement system in tumors and reviews recent research advances in the mechanisms of tumor immune tolerance and drug resistance.

## 1 Introduction

In recent years, remarkable progress has been made in advancing cancer treatment. However, the persistence of recurrence, metastasis, and drug resistance continues to be a huge hurdle in treating the cancer, making a considerable negative impact on patient outcome especially long-term survival. As a fundamental component of the human innate immune system, the complement system not only recognizes and eliminates pathogens directly but also participates in regulating adaptive immune responses, facilitating inflammatory responses, maintaining tissue stability, and repairing damages. Furthermore, the complement system plays a complex role in the occurrence and development of tumors, functioning as a double-edged sword (1, 2). Patients frequently present abnormal levels of certain complement components, while synthesis sites also demonstrate higher diversity and heterogeneity (3, 4). Complement components play a role in anti-tumor immunity through the complement dependent cytotoxicity (CDC) pathway, antibody-dependent cell-dependent cytotoxicity (ADCC), and antibody dependent cell mediated phagocytosis (ADCP), which can be considered the favorable side of the double-edged sword (5). In addition to liver and immune cells, cancer cells themselves are also capable of producing specific complement components (6). The aberrant expression of complement components has been demonstrated to have significant impact on the functionality of immune cells *in vivo*, as well as normal activation capacity of complement pathways and it’s overall immune responses to tumors, which represents the unfavorable side of this double-edged sword. The complex regulatory relationship between complement, cancer cells, and tumor microenvironment ultimately affects the outcome of tumor treatment (7). In conclusion, this article provides a concise overview of the composition and function of the complement system, especially those beneficial impact of complement system activation on tumors. Concurrently, this review conducts a comprehensive examination of its role in the development of immune tolerance and the mechanism behind tumor resistance. The objective is to synthesize and analyze the “double-edged sword” function of the complement system in tumor and establish a theoretical foundation for advancing novel immunotherapy strategies.

## 2 Overview of the complement system

### 2.1 Composition, activation and regulation

The complement system represents a fundamental component of the innate immune response, constituted with over 50 soluble proteins and membrane-binding components. These include complement component, complement activation products, complement receptors, complement regulatory proteins, and others (8). The synthesis of complement components is primarily carried out by the liver, while additional synthesis by other cell types could maximize the capacity, including mast cells, monocyte macrophages, dendritic cells, lymphocytes, and so forth (9). Cancer cells are also capable of producing specific complement components (6). The complement system is capable of recognizing immune complexes (ICS) via soluble pattern recognition receptors (PRRs) (10), pathogen-associated molecular patterns (PAMPs) (11) and damage-associated molecular patterns (DAMPs) (12, 13), triggering the complement cascade reaction (14).

The complement system comprises three principal activation pathways, namely the classical pathway (CP), the lectin pathway (LP), and the alternative pathway (AP) (15). The classical pathway is initiated by a robust interaction between complement component C1q and the Fc region of specific antibodies (predominantly IgM, IgG1, IgG2, and IgG3) present in immune complexes. This binding event serves as the stimulus for the activation of complement C1r. Additionally, C1s is activated in a cascade, collectively forming the C1 complex (C1qr2s2). Subsequently, the activated C1s enzyme catalyzes the cleavage of complement C4 and C2. Complement C4 is cleaved into C4a and C4b, while complement C2 is cleaved into C2a and C2b. The combination of C4b and C2a forms the C3 convertase C4bC2a (The C2a in the earliest C3 convertase C4bC2a refers to the larger molecular weight product of complement C2 cleavage products. In recent years, the academic community has named the large fragments of complement C2 decomposition products C2b, and therefore, C3 convertase has also been described as C4bC2b in some studies (16)). The lectin pathway is depended on mannose-binding lectin (MBL), a pattern recognition molecule that is capable of identifying and binding to carbohydrate ligands that are present on the surfaces of pathogens. Following this recognition, MBL attaches to mannose residues and recruits associated serine proteases, which are known as MASPs. The assembly of this MBL-MASPs complex is indicative of the activation of the lectin pathway (15). Conversely, the alternative activation pathway can be initiated spontaneously through hydrolysis of C3 molecules or when trace amounts of residual C3b are present. This process generates an active form, known as C3 convertase (C3bBb), which is distinctive from the other three activation pathways. Despite their separation, these pathways are interrelated. These pathways converge at the stages of cleaving C3 and forming C3 convertase, which jointly facilitate the formation of C5 convertase. The classical and lectin pathways form C4bC2aC3b, while the alternative pathway forms C3bBbC3b (17). Subsequently, complement C5 is cleaved into C5a and C5b under the action of C5 convertase. C5b, together with C6, C7, C8, and several C9 molecules, constitutes the membrane attack complex (MAC), which is responsible for the elimination of pathogens, the induction of inflammatory responses, and immune regulation (18). These three activation pathways, though independent, are interrelated and collectively constitute a sophisticated and efficacious immune defense network.

**Figure 1:**
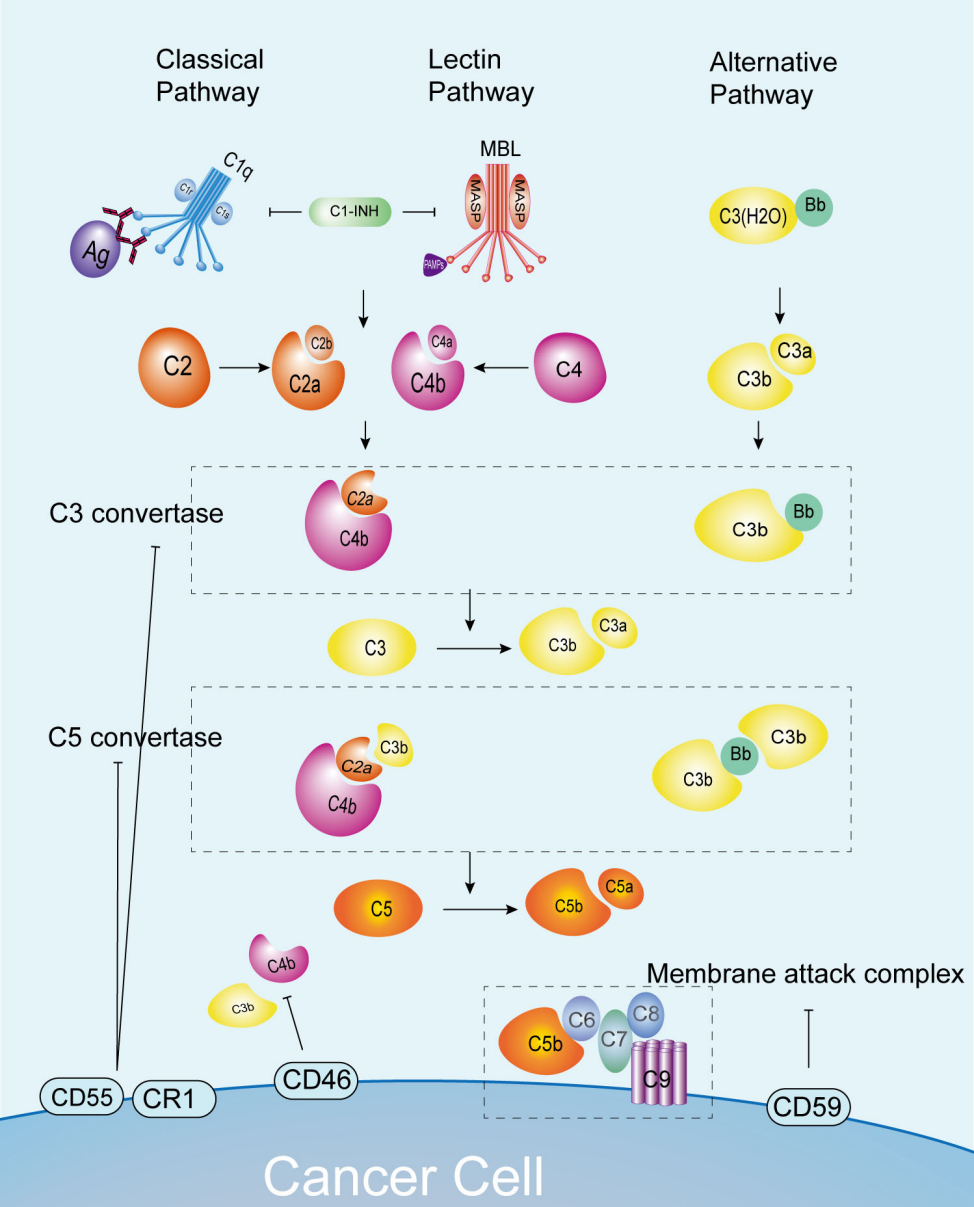
Complement pathways and normal anti-tumor immunity. C3 convertase and C5 convertase formation occurs via the classical, lectin, and alternative pathways, leading to C5 cleavage into C5a and C5b. This process enables the formation of MAC and cytotoxicity against cancer cells. Complement pathway activation is regulated by inhibitors like C1-INH, CD55, and CD46 to prevent overactivation. C1-INH, C1 inhibitor; CR1, complement receptor 1; MAC, membrane attack complex; MASP, MBL serine proteases; MAVS, mitochondrial anti-viral signaling protein; MBL, mannose-binding lectin.

The complement activation process is strictly controlled to ensure that its activity is limited to the surface of recognized pathogens, thereby avoiding damage to host cells and maintaining the immune balance of the system (19, 20). For instance, the C1 inhibitor (C1-INH) is capable of suppressing the activities of C1r, C1s, and MASP2. Additionally, C3b and C4b can be inactivated by serine protease complement factor I, CD46, complement receptor type 1 (CR1), fluid-phase Factor H, or C4b-binding protein (C4BP). The activity of the convertase is regulated by the decomposition of regulatory factors with decay-accelerating activity, including CD55, CR1, and C4BP. Additionally, the MAC is regulated by CD59 and vitronectin (S protein) (21). The activation of the complement system is subject to precise regulation by a range of complement inhibitors, complement regulatory proteins and cytokines. Abnormal activation is closely associated with a number of autoimmune and inflammatory diseases, including systemic lupus erythematosus and tubulointerstitial inflammation (22, 23) (**Figure 1**).

### 2.2 The complement system in intracellular and extracellular environment

For a considerable period, the complement system has been perceived as a straightforward bactericidal cascade mechanism, whereby the generation of membrane attack complexes (MACs) eliminates invading host bacteria (1). The activation of the complement pathway to form MACs and exert cytotoxic effects on target cells is defined as complement-dependent cytotoxicity (CDC) (24). Serum complement-activation fragments serve as key mediators of the immediate inflammatory response driven by innate immune cells, with the aim of curbing pathogen proliferation at the earliest stages (25). However, as the system has undergone extensive research, it is now widely acknowledged that complement not only is a complex innate immune surveillance system but also plays significant roles in host homeostasis (26), in adaptive immune regulation (27) and in the interaction between T cells and B cells (28).

Complement not only exerts its critical immunoregulatory function on cell membrane, but also plays an important regulatory role inside the cell. Within the internal structures of immune cells, including neutrophils, monocytes, T/B lymphocytes, complement components C3 and C5, and their activated fragments, can be detected in conjunction with corresponding receptors. These structures encompass the cytoplasm, lysosomes, endoplasmic reticulum, mitochondrial outer membrane, and the nucleus. Concurrently, specific complement regulatory proteins are also distributed in these regions, facilitating the precise adjustment of immune responses in cells (29). Furthermore, complement and its activated fragments, along with corresponding receptors, have been detected in diverse hematopoietic stem cells (30). These intracellular complement components are also designated as complosome, which primarily stems from two sources: the intracellular functionality of complement components synthesized by the cells themselves, and the endocytosis of exogenous complement components. The complosome is implicated in a number of fundamental cellular physiological processes, including cell metabolism (31–33), autophagy, and the regulation of gene expression (34). Kremlitzka et al. demonstrated that serum-derived and internalized C3 binds to the nucleus of human B cells and interacts with DNA, thereby participating in the regulation of gene transcription (35). Arbore et al. demonstrated that autocrine stimulation of the CYT-1 intracellular domain of CD46 is essential for the induction of amino acid transporter LAT1 expression and the enhancement of glucose transporter GLUT1 expression (32). CD46 exerts an influence on the cytotoxic function of CD8^+^ T cells, operating through its impact on fatty acid metabolism (36).

### 2.3 The anti-tumor mechanisms of the complement system

The complement system, which forms part of the body’s natural immune defenses, plays a crucial role in tumor immune surveillance. It is responsible for recognizing and eliminating infected cells or cancer cells that have abnormal antigen expression, by utilizing a range of sophisticated mechanisms. This process primarily encompasses several key pathways, including CDC, ADCC and antibody-dependent cell-mediated phagocytosis (ADCP) (5). Specifically, certain constituents of the complement system can tag tumor cells, facilitating the recognition and elimination of these tagged tumor cells by functional cells within the immune system. Additionally, complement components can augment the uptake and destruction of tumor cells by phagocytes via the process of opsonization, further strengthening the capacity of immune system to eliminate tumor cells (21). The anti-tumor effect of complement through CDC, ADCC and other pathways represents the pharmacological mechanism of classic monoclonal antibody treatments such as rituximab and daratumumab (37). Complement also plays an important guiding role in the development of novel anti-tumor targeted drugs, such as Claudin18.2 targeted antibodies (38). Editing the Fc segment of monoclonal antibodies and altering their ability in binding to complement C1q to alter the activation of the classical complement pathway is also an important strategy for current iterations (39).

## 3 The complement system and tumor immune tolerance

**Figure 2:**
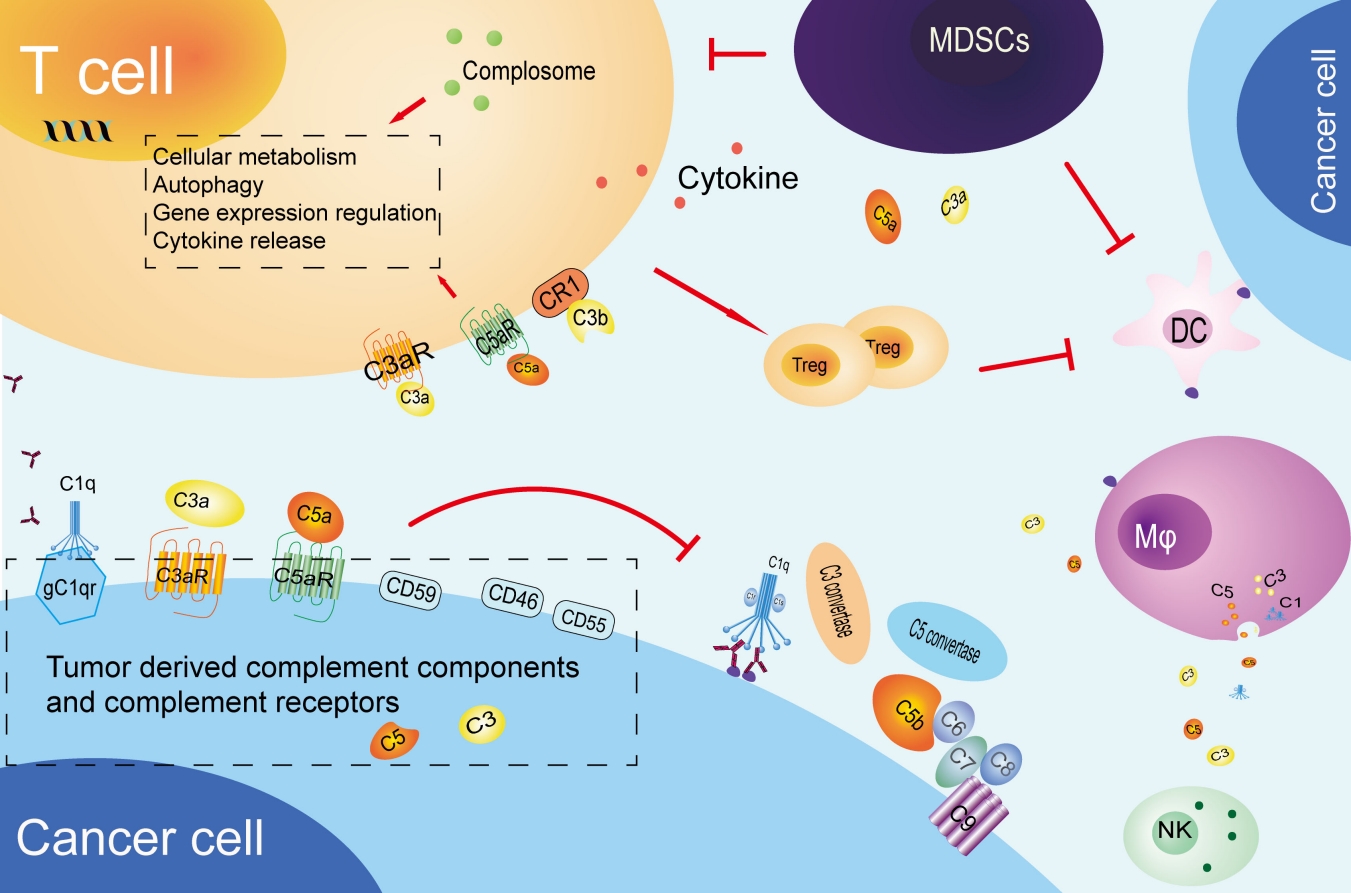
Tumor immune tolerance mediated by complement system. cancer cells produce complement components and inhibitory receptors that promote their growth and suppress immune response. These components influence T cells, promote Treg differentiation, and help establish an immune-suppressive environment. They also impair antigen-presenting cells, recruit MDSCs, and affect NK cell function and macrophage polarization. CR1, complement receptor 1; Mφ, macrophage; MDSCs, myeloid-derived suppressor cells; DC, Dendritic cells; NK, Natural killer cell.

The complement system plays a multifaceted role in the immune response to tumors. In addition to its anti-tumor function, the complement system is also involved in the formation of tumor immune tolerance. tumor immune tolerance can be defined as the phenomenon whereby tumor cells adapt to and resist the attack of the immune system, either directly or indirectly through alterations to the tumor microenvironment. This could be one of the triggers in promoting cancer cells growth and development. All parts of the complement system, including complement components, complement activation products, complement receptors, and complement regulatory proteins, are inextricably linked to the formation of immune tolerance. Cancer cells are capable of producing a range of complement components, which can facilitate immune evasion and self-promotion by interfering with immune cell function or impeding the normal complement cascade (**Figure 2**).

### 3.1 The complement system interferes with T cell function and promotes immune tolerance

We summarize the mechanisms by which the complement system participates in the formation of tumor immune tolerance by inhibiting T cell function into two categories. One is the direct action of complement lytic products on T cells, and the other one is the inhibition of T cell function by complement regulatory proteins expressed in tumors. The complement system can directly act on regulatory T cells (Tregs) through its cleavage products C3a and C5a, thereby affecting their activation and function. This mechanism is of critical importance for the maintenance of immune tolerance and the prevention of autoimmune reactions in the human body. C3a and C5a bind to corresponding receptors, thereby promoting the proliferation and activation of Treg cells and enhancing their immune suppression, including the promotion of anti-inflammatory cytokine interleukin-10 (IL-10) secretion (40). Torok et al. demonstrated that the activation of CD4^+^ T cell surface complement receptor 1 (CD35, CR1) expression induces these cells to transform into regulatory T cells (Tregs), not only enhancing the generation of IL-10 but also significantly reducing pro-inflammatory cytokine interferon-gamma (IFNγ) secretion (41). In studies of lung cancer, it was observed that C57BL/6J mice lacking complement C3 (C3-/- mice) exhibited markedly diminished growth of primary and metastatic tumors. This was associated with an increase in the number of CD4^+^ T cells expressing IFNγ^+^/TNFα^+^/IL-10^+^ and CD8^+^ T cells. The reversal of tumor suppression caused by the absence of complement C3, resulting from the immune exhaustion of CD4^+^ T cells, provides evidence that tumors can drive immune evasion through C3/C5-dependent pathways (42).

Complement regulatory proteins are a type of molecule that plays a pivotal role in regulating complement activation and related functions, particularly in the context of tumor immune regulation. The expression levels of complement regulatory proteins CD46, CD55, and CD59 are notably elevated in various tumors, including head and neck squamous cell carcinoma (HNSCC) (43), colon cancer, renal cancer, lung cancer (44), and breast cancer (45). The expression of complement regulatory proteins on the surface of T cells can directly participate in the regulation of T cell proliferation and differentiation. While CD46 does not impede the maturation of dendritic cells, it facilitates the differentiation of T cells into regulatory T cells (Treg cells) by stimulating the secretion of granulocyte-macrophage colony-stimulating factor (GM-CSF) and soluble tumor necrosis factor-related activator protein (CD40L). Although Treg cells do not inhibit the maturation of dendritic cells, they secrete IL-10, which suppresses the proliferation of bystander T cells. This maintains immune tolerance and prevents excessive immune responses (46). There is currently limited research on the mechanism of complement regulatory protein production in cancer cells. Shao et al. observed a negative correlation between the levels of CD55/CD59 and the infiltration of M1 macrophages and CD8^+^ T cells in human lung cancer specimens. Cancer cells upregulate the expression of CD55/CD59 through the EGFR/Wnt/β-catenin pathway, which results in the inhibition of the complement system and cytokine secretion. This ultimately leads to the suppression of CD8^+^ T cell activation, which in turn contributes to tumor immune evasion and resistance to immune checkpoint inhibitors. Inhibition of this pathway has been demonstrated to enhance the sensitivity of lung cancer to checkpoint inhibitors (47).

### 3.2 The complement system influences other immune cells in the tumor microenvironment

In the tumor microenvironment, the complement system has the capacity to regulate the antigen-presenting ability of immune cells other than T cells, thereby promoting the formation of tumor immune tolerance. Complement components, particularly C3a and C5a, influence the immune microenvironment by impacting the functionality of dendritic cells and other antigen-presenting cells. This, in turn, modulates the activation and antigen presentation of these cells to T cells (48–52). Complement components not only act directly on immune cells, but also indirectly inhibit the anti-tumor CD8^+^ T cell-mediated immune response by recruiting and enhancing the activity of myeloid-derived suppressor cells (MDSCs). In studies on a colorectal cancer model, the C5a/C5aR1 signaling pathway was identified as a factor promoting the accumulation of MDSCs in pathological colorectal tissue. This leads to impaired CD8^+^ T cell function and, consequently, to an increase in tumor cell growth, which is achieved by regulating the production of key cytokines and chemokines (53). Subsequent reports have indicated that C5aR1 expressed on MDSCs can also impair anti-tumor T cell responses by binding to ribosomal protein S19 (RPS19) (54). In the context of squamous cell carcinoma, C5a has been demonstrated to facilitate the pro-tumor characteristics of C5aR1^+^ mast cells and macrophages, whilst simultaneously inhibiting CD8^+^ T cell cytotoxicity and limiting chemotherapy-induced T cell responses, supporting the growth of tumors (55). The complement system regulates specific chemokines, such as chemokine CXC, which can control inflammatory responses and are closely related to angiogenesis and extracellular matrix remodeling required for tissue repair. These changes promote cancer cells infiltration and metastasis (56, 57). Researchers have found that the activation of C1q in ascites of peritoneal metastatic gastric cancer (PM-GC) patients may exacerbate the immune escape mechanism of tumors by promoting the accumulation of immunosuppressive cells in the tumor microenvironment (58).

### 3.3 Tumor-derived complement components inhibit tumor immunity

Cancer cells have the capacity to produce complement components or inhibitory receptors, which promote their own proliferation by self-secretion or by interfering with the normal function of complement in the microenvironment. This enables them to evade from immune surveillance and play a role similar to immune checkpoint receptors.

Studies have identified complement receptors C3aR and C5aR as a new class of immune checkpoint receptors. The production of the cytokine IL-10 can be blocked through complement signaling on effector T lymphocytes, which allows C3aR and C5aR expressed on effector T lymphocytes to inhibit the activation of immune cells (59). In lung cancer research, it was demonstrated that the expression of C5aR in tumor tissue was markedly elevated. Furthermore, Gcn5-mediated KLF5 acetylation facilitated GDF15 gene transcription and cell proliferation in lung cancer cells in response to C5a stimulation (60). In studies of ovarian and lung cancers, Cho et al. demonstrated that tumors are capable of deriving C3aR and C5aR, enhancing the survival of cancer cells through the activation of PI3K/AKT signaling pathways via self-secretion, rather than affecting T cell function. This is negatively correlated with the survival rate of ovarian cancer patients (61). Tumors are capable of producing specialized receptors that target complement, thereby enhancing cancer cells survival. One such receptor is the hepatitis B virus X protein (HBV X protein), which has been observed to upregulate CD46 expression, thereby enabling hepatocellular carcinoma cells and hepatocytes to evade from complement-mediated attack (62). Piao et al. discovered that colon cancer cell lines are capable of producing C5a in the absence of serum. Furthermore, they observed that C5a levels increased over time in a mouse model of colon cancer liver metastasis. Their findings suggest that blocking C5aR may reduce Mφ release of MCP-1 and, consequently, the ability of colon cancer cells to metastasize to the liver (63). Additionally, radiation therapy has been shown to promote the release of C5a by cancer cells and to upregulate C5a (64).

Cancer cells can also express C1q receptors gC1qR and cC1qR. These receptors exert a significant influence on tumor cell proliferation and survival, while may also facilitate tumor growth by regulating intercellular interactions within the microenvironment (65). gC1qR is highly expressed in various types of cancer cells and is associated with the Warburg effect, whereby cellular metabolic processes are converted from oxidative phosphorylation to glycolysis. Furthermore, gC1qR has been demonstrated to inhibit the mitochondrial permeability transition pore, thereby conferring protection against cellular damage induced by oxidative stress (66). A previous study revealed that C1q levels were diminished in patients with multiple myeloma (MM) during the disease progression stage (67). This finding may be associated with the inhibitory effect of gC1qR on C1q’s inhibitory effect on MM (68).

## 4 The complement system and tumor drug resistance

**Figure 3:**
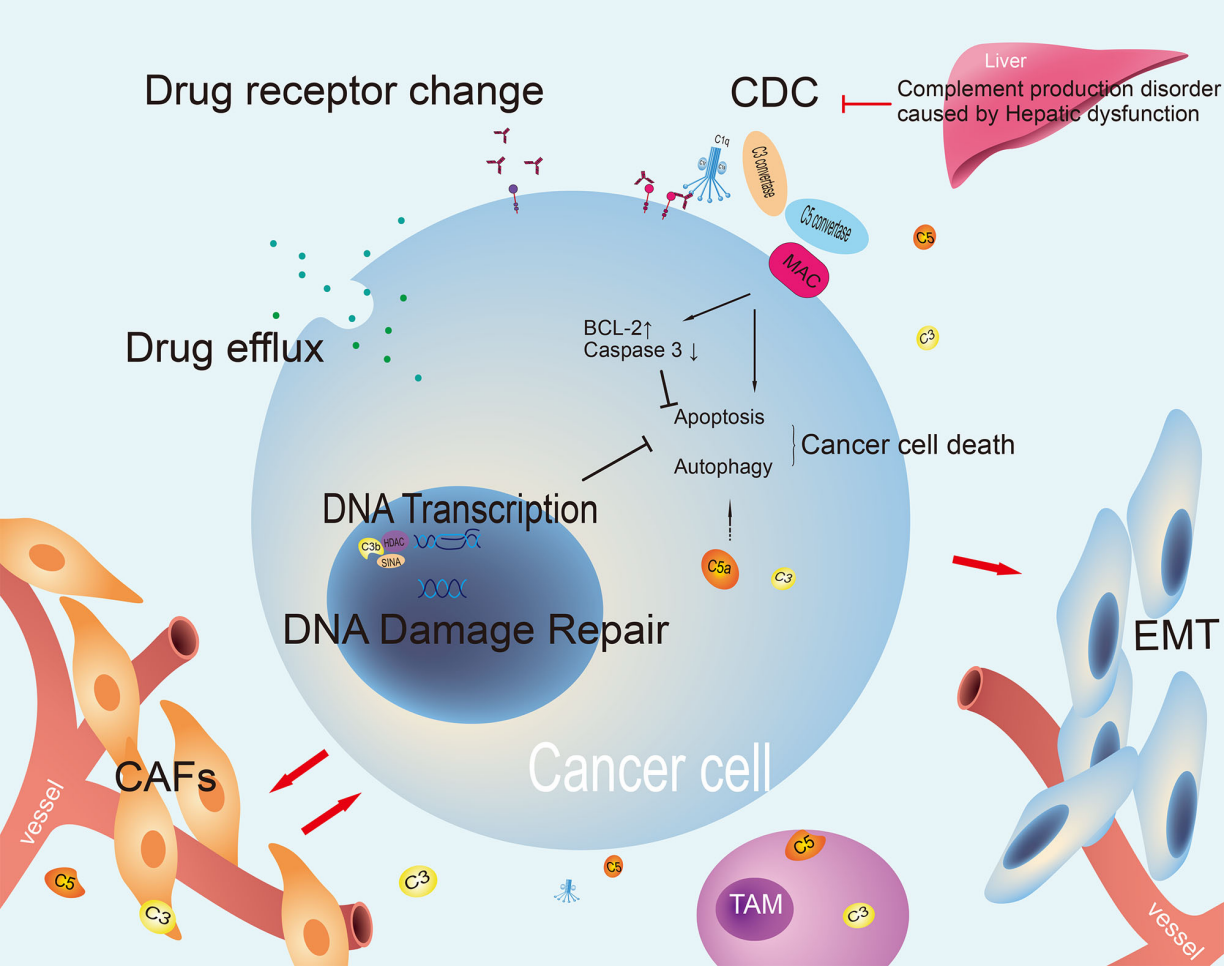
The mechanism of tumor treatment resistance involving complement. The complement pathway can both kill cancer cells and regulate processes that support cancer survival. It affects chemotherapy resistance and drug efflux. Changes in complement or drug targets can impact treatment effectiveness. It also interacts with the tumor microenvironment to promote cancer growth and EMT, indicating its complex role in cancer treatment resistance. CDC, complement dependent cytotoxicity; CAFs, cancer-associated fibroblasts; EMT, epithelial-mesenchymal transition; TAM, tumor-associated macrophage; MAC, membrane attack complex.

The mechanism of tumor drug resistance is complex and the presence of common factors, including those related to the intracellular and extracellular microenvironment, has been identified. Intracellular factors, including drug inactivation, alteration of drug targets, drug efflux, DNA damage repair, inhibition of cell death, epithelial-mesenchymal transition (EMT), intrinsic cellular heterogeneity, epigenetic effects, and inhibitory ligand formation, can influence the efficacy of pharmaceutical agents (69). The extracellular microenvironment may also exert an influence, including insufficient infiltration of T cells or inhibition of their function, expression of inhibitory immune checkpoints, and interference by MDSCs (70). Current research indicates that the complement system also plays a key role in tumor treatment resistance (**Figure 3**).

### 4.1 DNA transcription and DNA damage repair

Complement components have been demonstrated to influence DNA transcription and participate in the repair of DNA damage. In the study of non-small cell lung cancer models, it was discovered that C3b, produced by tumor cells, can translocate to the nucleus and interact with the SINA/HDAC complex, which plays a role in chromatin remodeling. This interaction forms the C3b/SIN3A/HDAC co-repressive complex. This complex facilitates histone 3 deacetylation at its promoter region, thereby suppressing the transcription of downstream target genes (such as GADD45A), inhibiting paclitaxel (PTX)-induced cell apoptosis and resulting in PTX resistance (71).

Glioblastoma-derived lnc-TALC can be encapsulated in exosomes and delivered to tumor-associated macrophages. In conjunction with complement C5/C5a produced by microglia, it facilitates macrophage polarization towards the M2 phenotype. Furthermore, complement C5 has been demonstrated to facilitate the repair of temozolomide (TMZ)-induced DNA damage, thereby contributing to chemotherapy resistance (72).

### 4.2 Cell death pathways

The components of the complement system have been demonstrated to play a crucial role in the regulation of cell apoptosis and autophagy. Furthermore, an association has been observed between these components and the malignant transformation of cells (34, 73). Under normal physiological conditions, the membrane MAC composed of C5b-9 can penetrate the cell membrane and trigger cell death (21, 74). However, the role of C5b-9 in oncology is more intricate. On one hand, following robust complement activation and sufficient membrane insertion of C5b-9 complexes, it can lead to the perforation of cell membranes and organelles, such as mitochondria (75). Research has identified elevated intracellular calcium ion concentrations and activated JNK, Bid, RIPK1, RIPK3, and MLKL as factors that can promote cell lysis (76). Conversely, cancer cells have been observed to release various protective measures to ensure their own survival (77). These include the blocking of complement activation through the action of complement regulatory proteins such as CD46, CD55, and CD59, thereby reducing the number of C5b-9 complexes inserted into the membrane, or through the action of caveolin (75). The inhibition of multiple pathways such as dynamin, Hsp70, Hsp90, PKC, and ERK has also been shown to promote the elimination of C5b-9 from the cell surface (78). Indeed, C5b-9 can be categorized as either lytic or sublytic, with lytic C5b-9 binding to the target cell membrane in a manner that results in the formation of MAC (79). The presence of complement limiting factors such as CD59, C3a, and MCP on the surface of the targeted cell results in the disruption of C5b-9 formation and the insertion of the phospholipid bilayer into the targeted cell. The membrane insertion effect of C5b-9 is shallow and cannot cause cell perforation and rupture. At this stage, the C5b-9 complex is referred to as sublytic C5b-9 (80). The effects of sublytic C5b-9 have been observed to be both anti-tumor and pro-tumor, with the former being the more predominant (81). However, it is widely accepted that the effects of sublytic C5b-9 tend to induce cell cycle acceleration, promote cell proliferation and survival by activating signaling pathways and transcription factors in cancer cells. For instance, sublytic C5b-9 has been observed to activate the PI3K/AKT/FOXO1 and ERK1 pathways in a Gi protein-dependent manner, thereby regulating the cell cycle (82). Furthermore, sublytic C5b-9 has been shown to promote the cell cycle by activating the complement response gene (RGC) -32 (83), which in turn activates the AKT and CDC2 kinase pathways (84). Furthermore, sublytic C5b-9 has been shown to inhibit the apoptosis of cancer cells, thereby promoting tumor growth, and drug resistance formation (82). This contributes to the growth, malignancy and formation of drug resistance of tumor.

Autophagy is an intracellular degradation and recycling process that is typically initiated when cells encounter nutrient deprivation or other forms of cellular stress. This process plays a crucial role in maintaining cellular homeostasis and survival (70). In cancer cells, autophagy is frequently regulated to support their growth and survival. Inhibition of the autophagy pathway has been demonstrated to enhance the efficacy of certain anti-tumor treatments (85). Wang et al. have demonstrated that C5a may exert an influence on the autophagy process of cells by regulating autophagy-related signaling pathways, including PI3K/AKT (86). This suggests that the complement system may plays a role in regulating cancer cells survival through autophagy-related pathways and may also be involved in drug resistance.

### 4.3 Epithelial-mesenchymal transition

A multiplicity of complement components are correlated with EMT. C3a/C3aR and transforming growth factor-beta (TGF-β synergistically act to facilitate the assembly and activation of NLRP3 inflammasome, thereby promoting the EMT of renal tubular epithelial cells via the ERK signaling pathway (87). Monocytes can be classified into three subtypes, namely classical types, intermediate, and non-classical types, based on the expression of CD14/CD16 (88). Researches have disclosed that the activation of classical and alternative complement pathways by C5a augments the production of CCL2 by PMCs, thereby facilitating the infiltration of intermediate monocytes into the microenvironment. Subsequently, the release of IL-1β by intermediate monocytes further boosts the proliferation, migration, adhesion, and EMT of cancer cells, while concurrently reducing the apoptosis rate of cancer cells.

### 4.4 Changes of the tumor microenvironment

Complement plays a pivotal role in regulating tumor resistance within the microenvironment, primarily through three key mechanisms, including the direct influence of complement content on CDC, the actions of complement on immune cells and the impacts of complement on non-immune cells within the microenvironment.

Monoclonal antibody therapy, which eradicates tumor cells through immune-mediated cell killing mechanisms, such as ADCC, CDC, and ADCP pathways, or by modulating T cell function through immune checkpoint blockade, has emerged as one of the most rapidly evolving and efficacious tumor treatment modalities (89). The levels of complement components in the microenvironment will inevitably affect the killing efficacy of CDC and the efficacy of CDC-dependent therapeutic agents such as anti-CD20 monoclonal antibodies and anti-CD38 monoclonal antibodies (90, 91). In the context of MM, the expression levels of CD55 and CD59 on the surface of MM cells were found to increase after resistance to daratumumab treatment. All trans retinoic acid (ATRA) reverses resistance to daratumumab by increasing the expression level of CD38 and decreasing the levels of CD55 and CD59 (92). NANOG is a key transcription factor that drives cancer cells towards multidrug resistance and stem cell-like phenotypes. Through the mechanism of promoter occupancy, it increases CD59, thereby enhancing cancer cells resistance to complement-dependent cytotoxicity (CDC) (93, 94). Targeting NANOG renders immune-refractory tumor cells sensitive to trastuzumab-mediated CDC (95). In short, understanding the mechanisms of complement component abnormalities, developing specific drugs or modifying treatment plans to maintain appropriate levels of complement components is a feasible way to improve treatment sensitivity.

In examining the influences exerted by immune cells within the microenvironment, it is evident that, in addition to the previously outlined T cell-related effects, the complement system plays a pivotal role in regulating the activities of tumor-associated macrophages (TAMs) and cancer-associated fibroblasts (CAFs). This in turn alters the tumor microenvironment, facilitating the growth and development of drug resistance in cancer cells. In particular, the activation of the C5a/C5aR signaling pathway has been demonstrated to significantly accelerate tumor progression in gastric cancer by regulating the PI3K/AKT signaling pathway and reducing the expression of p21. The pathway may contribute to enhanced resistance to treatment by altering the activities of TAMs and CAFs. This mechanism not only enhances the survival and proliferation capabilities of cancer cells but also promotes an immunosuppressive state within the tumor microenvironment by modulating the functions of immune cells, which in turn results in the formation of tumor resistance (96). Furthermore, macrophages that express uridylyl phosphate adenosine (uPA) regulate the non-C3-dependent release of C5a during the pre-neoplastic progression. This causes alterations in the pro-cancer characteristics of C5aR1^+^ mast cells and macrophages to inhibit the cytotoxicity of CD8^+^ T cells. It is also associated with paclitaxel chemotherapy resistance (55).

CAFs, as abundant and heterogeneous stromal cells in the tumor microenvironment, play a crucial role in the progression of cancer. Su et al. found that a subset of CAFs expressing both CD10 and GPR77 (CD10^+^GPR77^+^CAFs) is associated with chemotherapy resistance and low survival rates in cancer patients. CD10^+^GPR77^+^CAFs continuously activate the NF - κ B pathway through p65 phosphorylation and acetylation, providing a survival niche for cancer stem cells to promote tumor formation and chemotherapy resistance. This sustained activation is maintained by GPR77, which is a C5a receptor. Targeting these CAFs with anti-GPR77 neutralizing antibodies can eliminate tumor formation and restore tumor chemosensitivity (97). The C3aR signal plays a unique role in promoting lung metastasis of cancer by regulating CAFs. In the 4T1 tumor model, the production of pro metastatic cytokines by CAFs in C3aR knockout mice was significantly reduced. *In vitro* experiments have confirmed that the mechanism may be achieved by C3a-C3aR regulating the expression of TGF - β and CAF markers through the PI3K/AKT signaling pathway (98).

### 4.5 Drug efflux or modification of drug metabolism

The complement system exerts a significant influence on liver cell regeneration, with C3a and C5a being indispensable components during the process of liver regeneration (99). The prolonged use of chemotherapy in tumor patients has been shown to result in impaired liver and kidney function. This suggests that the capacity for complement synthesis may also be impaired, potentially influencing CDC-related functions. It seems plausible to suggest that complement components may be associated with drug excretion. Research has demonstrated that the basal complement sensitivity of arsenic trioxide-resistant melanoma cells was decreased by over 60%, and the amount of arsenic trioxide excretion was significantly increased, which represents a crucial factor in arsenic trioxide resistance (100). However, no direct evidence has been identified that the complement system directly participates in drug excretion or modifies drug metabolism.

## 5 The potential applications of complement regulators in overcoming treatment resistance

As the critical role of the complement system in tumor occurrence, development and treatment resistance is increasingly recognized, treatment strategies targeting complement are becoming a new trend in precision medicine for tumors. Currently, new drug development and treatment strategies targeting the complement system are beginning to take shape.

### 5.1 Development of novel complement-targeted therapeutics

Therapies targeting C3 and C5, the complement regulatory proteins CD46, CD55 and CD59, and complement inhibition strategies including the use of complement receptor antagonists can reshape the immune microenvironment and improve the sensitivity of conventional therapy, which are expected to become new treatment options to break through tumor drug resistance (101). Not only in chemotherapy and immunotherapy, but also in radiotherapy research, it has been found that C1-INH combined with radiotherapy can effectively improve the survival rate of subcutaneous glioblastoma animal models in mice. Although the survival of intracranial glioblastoma mouse models was not prolonged in this study, changing the dosage and route of administration of the drug in the future may provide ideas for improving efficacy in glioblastoma (102). However, current clinical research into the use of novel complement modulators is mainly focused on autoimmune diseases (103, 104). As the first complement inhibitor approved for the treatment of PNH and other complement-mediated diseases, eculizumab (105), its clinical application laid the foundation for the development of complement inhibitors (106). In addition, new complement inhibitors such as ravilizumab (long-acting C5 inhibitor) (107) and pegcetapolan (C3 inhibitor) have also been approved (108), demonstrating good efficacy and safety in the treatment of PNH. New monoclonal antibodies such as the C1s inhibitor sutimlimab (109) and the C5 inhibitor eculizumab are currently undergoing clinical trials in various autoimmune diseases and are expected to show good clinical results. However, there are currently no approved clinical trials of complement modulators in the adjuvant treatment of tumors.

### 5.2 Iterative strategy for monoclonal antibodies

The Fc segment of certain monoclonal antibody drugs binds to complement C1q molecules, activating the classical complement pathway and exerting tumor-killing effects via the CDC mechanism. However, there are significant differences in the dependence on the complement system and the intensity of CDC activation between different monoclonal antibodies. For example, different anti-CD20 antibodies have different abilities to activate complement, with ofamumab being the most effective, followed by rituximab, and obinutuzumab having a weaker binding ability (110, 111). The three drugs have also shown different clinical therapeutic effects in B-cell lymphoma (112). The CD38 monoclonal antibody daratumumab can effectively chelate hexavalent C1q and strongly activate the classical complement pathway by enhancing IgG1 dimerisation, resulting in effective killing of MM cells (113). However, whether complement C1q deficiency in MM patients affects this CDC-dependent cell killing effect remains to be determined in clinical studies. Modifying the Fc segment structure during the design phase of monoclonal antibodies and regulating their dependence on complement levels in patients may be a strategy to alter the stability of drug efficacy.

### 5.3 A novel treatment strategy combining complement

Research has shown that inhibiting both vascular endothelial growth factor (VEGF) and complement can significantly increase anti-tumor efficacy, especially in tumors that are resistant to anti-VEGF. This combination therapy strategy not only enhances cancer cells apoptosis but also inhibits angiogenesis. Future research can focus on how to optimize these combination therapies to maximize their clinical efficacy and reduce the drug resistance (114, 115).

Therefore, regulating the activity or block the complement system has the potential to reshape the immune microenvironment of tumors and increase the sensitivity of traditional treatments, offering new opportunities to address the problems of multiple drug resistance (2).

## 6 Concluding remarks

This review comprehensively reviews the composition and function of the complement system, outlines its role in anti-tumor therapy, and discusses the mechanism of complement components in the development of tumor immune tolerance. Recent researches and potential mechanisms of complement in tumor treatment resistance are also discussed. As the foundation of the innate immune system, complement exhibits complex and subtle dual properties during tumor progression, with both positive anti-tumor effects, it is also an important reason for tumors to establish immune escape and treatment resistance.

The complement system plays a dual role in tumors, but there are still many puzzles to be solved. Much remains to be understood about the complex dynamic network of interactions between the complement system, cancer cells and immune cells. Further research is needed to determine whether complement components modify metabolites, influence metabolic processes, and affect the metabolism of anti-tumor drugs such as chemotherapeutics and monoclonal antibodies by altering the metabolic capacity of the liver and kidneys. There is still a lack of basic and clinical research data on whether synchronous treatment with complement supplements or inhibitors can improve tolerance to tumor immune therapy, increase sensitivity to drug treatment or reverse drug resistance.

In summary, the importance of the complement system in tumourigenesis and drug resistance mechanisms is increasingly evident, providing us with new research directions and potential for clinical applications. However, how to balance the duality of complement in tumors still requires the joint efforts of medical researchers. In the face of this challenge, we look forward to further uncovering the complexity of the complement system in future research, thereby advancing personalized tumor treatment.

## References

[1] Mastellos DC Hajishengallis G Lambris JD. A guide to complement biology, pathology and therapeutic opportunity. Nat Rev Immunol. (2024) 24:118–41. doi: 10.1038/s41577-023-00926-1 37670180

[2] West EE Woodruff T Fremeaux-Bacchi V Kemper C. Complement in human disease: approved and up-and-coming therapeutics. Lancet. (2024) 403:392–405. doi: 10.1016/S0140-6736(23)01524-6 37979593 PMC10872502

[3] Luo S Wang M Wang H Hu D Zipfel PF Hu Y. How does complement affect hematological Malignancies: from basic mechanisms to clinical application. Front Immunol. (2020) 11:593610. doi: 10.3389/fimmu.2020.593610 33193442 PMC7658260

[4] Kolev M Das M Gerber M Baver S Deschatelets P Markiewski MM. Inside-out of complement in cancer. Front Immunol. (2022) 13:931273. doi: 10.3389/fimmu.2022.931273 35860237 PMC9291441

[5] Reis ES Mastellos DC Ricklin D Mantovani A Lambris JD. Complement in cancer: untangling an intricate relationship. Nat Rev Immunol. (2018) 18:5–18. doi: 10.1038/nri.2017.97 28920587 PMC5816344

[6] Afshar-Kharghan V. The role of the complement system in cancer. J Clin Invest. (2017) 127:780–89. doi: 10.1172/JCI90962 PMC5330758 28248200

[7] Roumenina LT Daugan MV Petitprez F Sautes-Fridman C Fridman WH. Context-dependent roles of complement in cancer. Nat Rev Cancer. (2019) 19:698–715. doi: 10.1038/s41568-019-0210-0 31666715

[8] Kemper C Pangburn MK Fishelson Z. Complement nomenclature 2014. Mol Immunol. (2014) 61:56–8. doi: 10.1016/j.molimm.2014.07.004 25081089

[9] Lubbers R van Essen MF van Kooten C Trouw LA. Production of complement components by cells of the immune system. Clin Exp Immunol. (2017) 188:183–94. doi: 10.1111/cei.12952 PMC5383442 28249350

[10] Broz P Monack DM. Newly described pattern recognition receptors team up against intracellular pathogens. Nat Rev Immunol. (2013) 13:551–65. doi: 10.1038/nri3479 23846113

[11] Garred P Tenner AJ Mollnes TE. Therapeutic targeting of the complement system: from rare diseases to pandemics. Pharmacol Rev. (2021) 73:792–827. doi: 10.1124/pharmrev.120.000072 33687995 PMC7956994

[12] Barichello T Generoso JS Singer M Dal-Pizzol F. Biomarkers for sepsis: more than just fever and leukocytosis-a narrative review. Crit Care. (2022) 26:14. doi: 10.1186/s13054-021-03862-5 34991675 PMC8740483

[13] Relja B Land WG. Damage-associated molecular patterns in trauma. Eur J Trauma Emerg Surg. (2020) 46:751–75. doi: 10.1007/s00068-019-01235-w PMC7427761 31612270

[14] Iwasaki A Medzhitov R. Regulation of adaptive immunity by the innate immune system. Science. (2010) 327:291–95. doi: 10.1126/science.1183021 PMC3645875 20075244

[15] Monach PA. Complement. Arthritis Rheumatol. (2024) 76:1–08. doi: 10.1002/art.42671 37551641

[16] Ricklin D Hajishengallis G Yang K Lambris JD. Complement: a key system for immune surveillance and homeostasis. Nat Immunol. (2010) 11:785–97. doi: 10.1038/ni.1923 PMC2924908 20720586

[17] Zipfel PF Skerka C. Complement regulators and inhibitory proteins. Nat Rev Immunol. (2009) 9:729–40. doi: 10.1038/nri2620 19730437

[18] Dunkelberger JR Song WC. Complement and its role in innate and adaptive immune responses. Cell Res. (2010) 20:34–50. doi: 10.1038/cr.2009.139 20010915

[19] Manrique-Caballero CL Peerapornratana S Formeck C Del RG Gomez DH Kellum JA. Typical and atypical hemolytic uremic syndrome in the critically ill. Crit Care Clin. (2020) 36:333–56. doi: 10.1016/j.ccc.2019.11.004 32172817

[20] Coss SL Zhou D Chua GT Aziz RA Hoffman RP Wu YL. The complement system and human autoimmune diseases. J Autoimmun. (2023) 137:102979. doi: 10.1016/j.jaut.2022.102979 36535812 PMC10276174

[21] Merle NS Church SE Fremeaux-Bacchi V Roumenina LT. Complement system part I - molecular mechanisms of activation and regulation. Front Immunol. (2015) 6:262. doi: 10.3389/fimmu.2015.00262 26082779 PMC4451739

[22] Ni H Pan W Jin Q Xie Y Zhang N Chen K. Label-free proteomic analysis of serum exosomes from paroxysmal atrial fibrillation patients. Clin Proteomics. (2021) 18:1. doi: 10.1186/s12014-020-09304-8 33407078 PMC7789314

[23] Panwar HS Ojha H Ghosh P Barage SH Raut S Sahu A. Molecular engineering of an efficient four-domain DAF-MCP chimera reveals the presence of functional modularity in RCA proteins. Proc Natl Acad Sci U.S.A. (2019) 116:9953–58. doi: 10.1073/pnas.1818573116 PMC6525521 31036650

[24] Oka T Matsuzawa Y Tsuneyoshi M Nakamura Y Aoshima K Tsugawa H. Multiomics analysis to explore blood metabolite biomarkers in an Alzheimer’s Disease Neuroimaging Initiative cohort. Sci Rep. (2024) 14:6797. doi: 10.1038/s41598-024-56837-1 38565541 PMC10987653

[25] Freeley S Kemper C Le Friec G. The “ins and outs” of complement-driven immune responses. Immunol Rev. (2016) 274:16–32. doi: 10.1111/imr.12472 27782335 PMC5102160

[26] Ayano M Horiuchi T. Complement as a biomarker for systemic lupus erythematosus. Biomolecules. (2023) 13:367. doi: 10.3390/biom13020367 36830735 PMC9953581

[27] Kulkarni HS. Hexamerization: explaining the original sin of IgG-mediated complement activation in acute lung injury. J Clin Invest. (2024) 134:e181137. doi: 10.1172/JCI181137 38828725 PMC11142731

[28] Ricklin D Reis ES Lambris JD. Complement in disease: a defence system turning offensive. Nat Rev Nephrol. (2016) 12:383–401. doi: 10.1038/nrneph.2016.70 27211870 PMC4974115

[29] West EE Kemper C. Complosome - the intracellular complement system. Nat Rev Nephrol. (2023) 19:426–39. doi: 10.1038/s41581-023-00704-1 PMC10100629 37055581

[30] Bujko K Brzenziakiewicz-Janus K Kucia M Ratajczak MZ. Intracellular complement (Complosome) is expressed in several types of human adult bone marrow-derived stem cells. Stem Cell Rev Rep. (2024) 20:437–39. doi: 10.1007/s12015-023-10650-x 37917411

[31] Ratajczak MZ Adamiak M Abdelbaset-Ismail A Bujko K Thapa A Chumak V. Intracellular complement (complosome) is expressed in hematopoietic stem/progenitor cells (HSPCs) and regulates cell trafficking, metabolism and proliferation in an intracrine Nlrp3 inflammasome-dependent manner. Leukemia. (2023) 37:1401–05. doi: 10.1038/s41375-023-01894-0 PMC10244163 37055506

[32] Kolev M Dimeloe S Le Friec G Navarini A Arbore G Povoleri GA. Complement regulates nutrient influx and metabolic reprogramming during th1 cell responses. Immunity. (2015) 42:1033–47. doi: 10.1016/j.immuni.2015.05.024 PMC4518498 26084023

[33] Chen X Zhao Z Jiang X Li J Miao F Yu H. The complement component 4 binding protein alpha gene: A versatile immune gene that influences lipid metabolism in bovine mammary epithelial cell lines. Int J Mol Sci. (2024) 25:2375. doi: 10.3390/ijms25042375 38397050 PMC10889797

[34] King BC Kulak K Krus U Rosberg R Golec E Wozniak K. Complement Component C3 Is Highly Expressed in Human Pancreatic Islets and Prevents beta Cell Death via ATG16L1 Interaction and Autophagy Regulation. Cell Metab. (2019) 29:202–10. doi: 10.1016/j.cmet.2018.09.009 30293775

[35] Kremlitzka M Nowacka AA Mohlin FC Bompada P De Marinis Y Blom AM. Interaction of serum-derived and internalized C3 with DNA in human B cells-A potential involvement in regulation of gene transcription. Front Immunol. (2019) 10:493. doi: 10.3389/fimmu.2019.00493 30941132 PMC6433827

[36] Arbore G West EE Rahman J Le Friec G Niyonzima N Pirooznia M. Complement receptor CD46 co-stimulates optimal human CD8(+) T cell effector function via fatty acid metabolism. Nat Commun. (2018) 9:4186. doi: 10.1038/s41467-018-06706-z 30305631 PMC6180132

[37] Toledo-Stuardo K Ribeiro CH Gonzalez-Herrera F Matthies DJ Le Roy MS Dietz-Vargas C. Therapeutic antibodies in oncology: an immunopharmacological overview. Cancer Immunol Immunother. (2024) 73:242. doi: 10.1007/s00262-024-03814-2 39358613 PMC11448508

[38] Xu G Liu W Wang Y Wei X Liu F He Y. CMG901, a Claudin18.2-specific antibody-drug conjugate, for the treatment of solid tumors. Cell Rep Med. (2024) 5:101710. doi: 10.1016/j.xcrm.2024.101710 39232496 PMC11528232

[39] van der Horst HJ Mutis T. Enhancing Fc-mediated effector functions of monoclonal antibodies: The example of HexaBodies. Immunol Rev. (2024) 328:456–65. doi: 10.1111/imr.13394 PMC11659923 39275983

[40] van der Touw W Cravedi P Kwan WH Paz-Artal E Merad M Heeger PS. Cutting edge: Receptors for C3a and C5a modulate stability of alloantigen-reactive induced regulatory T cells. J Immunol. (2013) 190:5921–25. doi: 10.4049/jimmunol.1300847 PMC3679341 23690475

[41] Torok K Dezso B Bencsik A Uzonyi B Erdei A. Complement receptor type 1 (CR1/CD35) expressed on activated human CD4+ T cells contributes to generation of regulatory T cells. Immunol Lett. (2015) 164:117–24. doi: 10.1016/j.imlet.2015.02.009 25742728

[42] Kwak JW Laskowski J Li HY McSharry MV Sippel TR Bullock BL. Complement activation via a C3a receptor pathway alters CD4(+) T lymphocytes and mediates lung cancer progression. Cancer Res. (2018) 78:143–56. doi: 10.1158/0008-5472.CAN-17-0240 PMC5810934 29118090

[43] Kesselring R Thiel A Pries R Fichtner-Feigl S Brunner S Seidel P. The complement receptors CD46, CD55 and CD59 are regulated by the tumour microenvironment of head and neck cancer to facilitate escape of complement attack. Eur J Cancer. (2014) 50:2152–61. doi: 10.1016/j.ejca.2014.05.005 24915776

[44] Niehans GA Cherwitz DL Staley NA Knapp DJ Dalmasso AP. Human carcinomas variably express the complement inhibitory proteins CD46 (membrane cofactor protein), CD55 (decay-accelerating factor), and CD59 (protectin). Am J Pathol. (1996) 149:129–42. PMC1865231 8686736

[45] Maciejczyk A Szelachowska J Szynglarewicz B Szulc R Szulc A Wysocka T. CD46 Expression is an unfavorable prognostic factor in breast cancer cases. Appl Immunohistochem Mol Morphol. (2011) 19:540–46. doi: 10.1097/PAI.0b013e31821a0be9 21617523

[46] Barchet W Price JD Cella M Colonna M MacMillan SK Cobb JP. Complement-induced regulatory T cells suppress T-cell responses but allow for dendritic-cell maturation. Blood. (2006) 107:1497–504. doi: 10.1182/blood-2005-07-2951 PMC1895395 16239430

[47] Shao F Gao Y Wang W He H Xiao L Geng X. Silencing EGFR-upregulated expression of CD55 and CD59 activates the complement system and sensitizes lung cancer to checkpoint blockade. Nat Cancer. (2022) 3:1192–210. doi: 10.1038/s43018-022-00444-4 36271172

[48] Vachino G Gelfand JA Atkins MB Tamerius JD Demchak P Mier JW. Complement activation in cancer patients undergoing immunotherapy with interleukin-2 (IL-2): binding of complement and C-reactive protein by IL-2-activated lymphocytes. Blood. (1991) 78:2505–13. doi: 10.1182/blood.V78.10.2505.2505 1824247

[49] Ajona D Ortiz-Espinosa S Pio R. Complement anaphylatoxins C3a and C5a: Emerging roles in cancer progression and treatment. Semin Cell Dev Biol. (2019) 85:153–63. doi: 10.1016/j.semcdb.2017.11.023 29155219

[50] Magrini E Minute L Dambra M Garlanda C. Complement activation in cancer: Effects on tumor-associated myeloid cells and immunosuppression. Semin Immunol. (2022) 60:101642. doi: 10.1016/j.smim.2022.101642 35842274

[51] Sayegh ET Bloch O Parsa AT. Complement anaphylatoxins as immune regulators in cancer. Cancer Med. (2014) 3:747–58. doi: 10.1002/cam4.241 PMC4303144 24711204

[52] Weitz IC. Thrombotic microangiopathy in cancer. Semin Thromb Hemost. (2019) 45:348–53. doi: 10.1055/s-0039-1687893 31041804

[53] Ding P Li L Li L Lv X Zhou D Wang Q. C5aR1 is a master regulator in Colorectal Tumorigenesis via Immune modulation. Theranostics. (2020) 10:8619–32. doi: 10.7150/thno.45058 PMC7392014 32754267

[54] Markiewski MM Vadrevu SK Sharma SK Chintala NK Ghouse S Cho JH. The ribosomal protein S19 suppresses antitumor immune responses via the complement C5a receptor 1. J Immunol. (2017) 198:2989–99. doi: 10.4049/jimmunol.1602057 PMC5360500 28228558

[55] Medler TR Murugan D Horton W Kumar S Cotechini T Forsyth AM. Complement C5a fosters squamous carcinogenesis and limits T cell response to chemotherapy. Cancer Cell. (2018) 34:561–78. doi: 10.1016/j.ccell.2018.09.003 PMC6246036 30300579

[56] Romagnani P Lasagni L Annunziato F Serio M Romagnani S. CXC chemokines: the regulatory link between inflammation and angiogenesis. Trends Immunol. (2004) 25:201–09. doi: 10.1016/j.it.2004.02.006 15039047

[57] Daugan MV Revel M Russick J Dragon-Durey MA Gaboriaud C Robe-Rybkine T. Complement C1s and C4d as prognostic biomarkers in renal cancer: emergence of noncanonical functions of C1s. Cancer Immunol Res. (2021) 9:891–908. doi: 10.1158/2326-6066.CIR-20-0532 34039653

[58] Li L Ding P Dong Y Shen S Lv X Yu J. CG001, a C3b-targeted complement inhibitor, blocks 3 complement pathways: development and preclinical evaluation. Blood Adv. (2024) 8:4181–93. doi: 10.1182/bloodadvances.2024012874 PMC11334799 38865712

[59] Wang Y Zhang H He YW. The complement receptors C3aR and C5aR are a new class of immune checkpoint receptor in cancer immunotherapy. Front Immunol. (2019) 10:1574. doi: 10.3389/fimmu.2019.01574 31379815 PMC6658873

[60] Zhao C Li Y Qiu W He F Zhang W Zhao D. C5a induces A549 cell proliferation of non-small cell lung cancer via GDF15 gene activation mediated by GCN5-dependent KLF5 acetylation. Oncogene. (2018) 37:4821–37. doi: 10.1038/s41388-018-0298-9 PMC6117268 29773900

[61] Cho MS Vasquez HG Rupaimoole R Pradeep S Wu S Zand B. Autocrine effects of tumor-derived complement. Cell Rep. (2014) 6:1085–95. doi: 10.1016/j.celrep.2014.02.014 PMC4084868 24613353

[62] Zhang S Shan C Cui W You X Du Y Kong G. Hepatitis B virus X protein protects hepatoma and hepatic cells from complement-dependent cytotoxicity by up-regulation of CD46. FEBS Lett. (2013) 587:645–51. doi: 10.1016/j.febslet.2013.01.019 23391762

[63] Piao C Cai L Qiu S Jia L Song W Du J. Complement 5a enhances hepatic metastases of colon cancer via monocyte chemoattractant protein-1-mediated inflammatory cell infiltration. J Biol Chem. (2015) 290:10667–76. doi: 10.1074/jbc.M114.612622 PMC4409234 25739439

[64] Yuan M Wang C Wu Y Qiao L Deng G Liang N. Targeting complement C5a to improve radiotherapy sensitivity in non-small cell lung cancer. Transl Lung Cancer Res. (2023) 12:1093–107. doi: 10.21037/tlcr-23-258 PMC10261863 37323177

[65] Ghebrehiwet B Zaniewski M Fernandez A DiGiovanni M Reyes TN Ji P. The C1q and gC1qR axis as a novel checkpoint inhibitor in cancer. Front Immunol. (2024) 15:1351656. doi: 10.3389/fimmu.2024.1351656 38711524 PMC11070495

[66] Sunderhauf A Raschdorf A Hicken M Schlichting H Fetzer F Brethack AK. GC1qR cleavage by caspase-1 drives aerobic glycolysis in tumor cells. Front Oncol. (2020) 10:575854. doi: 10.3389/fonc.2020.575854 33102234 PMC7556196

[67] Yang R Huang J Ma H Li S Gao X Liu Y. Is complement C1q a potential marker for tumor burden and immunodeficiency in multiple myeloma? Leuk Lymphoma. (2019) 60:1812–18. doi: 10.1080/10428194.2018.1543883 30628497

[68] Xu J Sun Y Jiang J Xu Z Li J Xu T. Globular C1q receptor (gC1qR/p32/HABP1) suppresses the tumor-inhibiting role of C1q and promotes tumor proliferation in 1q21-amplified multiple myeloma. Front Immunol. (2020) 11:1292. doi: 10.3389/fimmu.2020.01292 32760394 PMC7372013

[69] Housman G Byler S Heerboth S Lapinska K Longacre M Snyder N. Drug resistance in cancer: an overview. Cancers (Basel). (2014) 6:1769–92. doi: 10.3390/cancers6031769 PMC4190567 25198391

[70] Jiang T Chen X Ren X Yang JM Cheng Y. Emerging role of autophagy in anti-tumor immunity: Implications for the modulation of immunotherapy resistance. Drug Resist Update. (2021) 56:100752. doi: 10.1016/j.drup.2021.100752 33765484

[71] Wang X Hao Y Chen J Ding P Lv X Zhou D. Nuclear complement C3b promotes paclitaxel resistance by assembling the SIN3A/HDAC1/2 complex in non-small cell lung cancer. Cell Death Dis. (2023) 14:351. doi: 10.1038/s41419-023-05869-y 37291119 PMC10250389

[72] Li Z Meng X Wu P Zha C Han B Li L. Glioblastoma cell-derived lncRNA-containing exosomes induce microglia to produce complement C5, promoting chemotherapy resistance. Cancer Immunol Res. (2021) 9:1383–99. doi: 10.1158/2326-6066.CIR-21-0258 34667108

[73] Lin L Rodrigues F Kary C Contet A Logan M Baxter R. Complement-related regulates autophagy in neighboring cells. Cell. (2017) 170:158–71. doi: 10.1016/j.cell.2017.06.018 PMC5533186 28666117

[74] Nauta AJ Daha MR Tijsma O van de Water B Tedesco F Roos A. The membrane attack complex of complement induces caspase activation and apoptosis. Eur J Immunol. (2002) 32:783–92. doi: 10.1002/1521-4141(200203)32:3<783::AID-IMMU783>3.0.CO;2-Q 11870622

[75] Fishelson Z Kirschfink M. Complement C5b-9 and cancer: mechanisms of cell damage, cancer counteractions, and approaches for intervention. Front Immunol. (2019) 10:752. doi: 10.3389/fimmu.2019.00752 31024572 PMC6467965

[76] Thielen A van Baarsen IM Jongsma ML Zeerleder S Spaapen RM Wouters D. CRISPR/Cas9 generated human CD46, CD55 and CD59 knockout cell lines as a tool for complement research. J Immunol Methods. (2018) 456:15–22. doi: 10.1016/j.jim.2018.02.004 29447841

[77] Christmas SE de la Mata EC Halliday D Buxton CA Cummerson JA Johnson PM. Levels of expression of complement regulatory proteins CD46, CD55 and CD59 on resting and activated human peripheral blood leucocytes. Immunology. (2006) 119:522–28. doi: 10.1111/j.1365-2567.2006.02467.x PMC2265819 16999828

[78] Viedt C Shen W Fei J Kamimura M Hansch GM Katus HA. HMG-CoA reductase inhibition reduces the proinflammatory activation of human vascular smooth muscle cells by the terminal complement factor C5b-9. Basic Res Cardiol. (2003) 98:353–61. doi: 10.1007/s00395-003-0437-4 14556080

[79] Tegla CA Cudrici C Patel S Trippe RR Rus V Niculescu F. Membrane attack by complement: the assembly and biology of terminal complement complexes. Immunol Res. (2011) 51:45–60. doi: 10.1007/s12026-011-8239-5 21850539 PMC3732183

[80] Pippin JW Durvasula R Petermann A Hiromura K Couser WG Shankland SJ. DNA damage is a novel response to sublytic complement C5b-9-induced injury in podocytes. J Clin Invest. (2003) 111:877–85. doi: 10.1172/JCI15645 PMC153762 12639994

[81] Serna M Giles JL Morgan BP Bubeck D. Structural basis of complement membrane attack complex formation. Nat Commun. (2016) 7:10587. doi: 10.1038/ncomms10587 26841837 PMC4743022

[82] Vlaicu SI Tatomir A Rus V Rus H. Role of C5b-9 and RGC-32 in cancer. Front Immunol. (2019) 10:1054. doi: 10.3389/fimmu.2019.01054 31156630 PMC6530392

[83] Fosbrink M Cudrici C Tegla CA Soloviova K Ito T Vlaicu S. Response gene to complement 32 is required for C5b-9 induced cell cycle activation in endothelial cells. Exp Mol Pathol. (2009) 86:87–94. doi: 10.1016/j.yexmp.2008.12.005 19162005 PMC2699899

[84] Vlaicu SI Tegla CA Cudrici CD Danoff J Madani H Sugarman A. Role of C5b-9 complement complex and response gene to complement-32 (RGC-32) in cancer. Immunol Res. (2013) 56:109–21. doi: 10.1007/s12026-012-8381-8 23247987

[85] Debnath J Gammoh N Ryan KM. Autophagy and autophagy-related pathways in cancer. Nat Rev Mol Cell Biol. (2023) 24:560–75. doi: 10.1038/s41580-023-00585-z PMC9980873 36864290

[86] Wang L Sun Y Kong F Jiang Y An M Jin B. Mild hypothermia alleviates complement C5a-induced neuronal autophagy during brain ischemia-reperfusion injury after cardiac arrest. Cell Mol Neurobiol. (2023) 43:1957–74. doi: 10.1007/s10571-022-01275-8 PMC11412180 36006573

[87] You D Nie K Wu X Weng M Yang L Chen Y. C3a/C3aR synergies with TGF-beta to promote epithelial-mesenchymal transition of renal tubular epithelial cells via the activation of the NLRP3 inflammasome. J Transl Med. (2023) 21:904. doi: 10.1186/s12967-023-04764-6 38082306 PMC10714586

[88] Ravenhill BJ Soday L Houghton J Antrobus R Weekes MP. Comprehensive cell surface proteomics defines markers of classical, intermediate and non-classical monocytes. Sci Rep. (2020) 10:4560. doi: 10.1038/s41598-020-61356-w 32165698 PMC7067879

[89] Lee W Lee SM Jung ST. Unlocking the power of complement-dependent cytotoxicity: engineering strategies for the development of potent therapeutic antibodies for cancer treatments. Biodrugs. (2023) 37:637–48. doi: 10.1007/s40259-023-00618-1 37486566

[90] Felberg A Taszner M Urban A Majeranowski A Jaskula K Jurkiewicz A. Monitoring of the complement system status in patients with B-cell Malignancies treated with rituximab. Front Immunol. (2020) 11:584509. doi: 10.3389/fimmu.2020.584509 33329558 PMC7710700

[91] Saltarella I Desantis V Melaccio A Solimando AG Lamanuzzi A Ria R. Mechanisms of resistance to anti-CD38 daratumumab in multiple myeloma. Cells. (2020) 9:167. doi: 10.3390/cells9010167 31936617 PMC7017193

[92] Nijhof IS Casneuf T van Velzen J van Kessel B Axel AE Syed K. CD38 expression and complement inhibitors affect response and resistance to daratumumab therapy in myeloma. Blood. (2016) 128:959–70. doi: 10.1182/blood-2016-03-703439 27307294

[93] Song KH Kim JH Lee YH Bae HC Lee HJ Woo SR. Mitochondrial reprogramming via ATP5H loss promotes multimodal cancer therapy resistance. J Clin Invest. (2018) 128:4098–114. doi: 10.1172/JCI96804 PMC6118592 30124467

[94] Song KH Oh SJ Kim S Cho H Lee HJ Song JS. HSP90A inhibition promotes anti-tumor immunity by reversing multi-modal resistance and stem-like property of immune-refractory tumors. Nat Commun. (2020) 11:562. doi: 10.1038/s41467-019-14259-y 31992715 PMC6987099

[95] Son SW Cho E Cho H Woo SR Lee HJ Oh SJ. NANOG confers resistance to complement-dependent cytotoxicity in immune-edited tumor cells through up-regulating CD59. Sci Rep. (2022) 12:8652. doi: 10.1038/s41598-022-12692-6 35606403 PMC9126891

[96] Chen J Li GQ Zhang L Tang M Cao X Xu GL. Complement C5a/C5aR pathway potentiates the pathogenesis of gastric cancer by down-regulating p21 expression. Cancer Lett. (2018) 412:30–6. doi: 10.1016/j.canlet.2017.10.003 29031586

[97] Su S Chen J Yao H Liu J Yu S Lao L. CD10(+)GPR77(+) cancer-associated fibroblasts promote cancer formation and chemoresistance by sustaining cancer stemness. Cell. (2018) 172:841–56. doi: 10.1016/j.cell.2018.01.009 29395328

[98] Shu C Zha H Long H Wang X Yang F Gao J. C3a-C3aR signaling promotes breast cancer lung metastasis via modulating carcinoma associated fibroblasts. J Exp Clin Cancer Res. (2020) 39:11. doi: 10.1186/s13046-019-1515-2 31931851 PMC6958674

[99] Strey CW Markiewski M Mastellos D Tudoran R Spruce LA Greenbaum LE. The proinflammatory mediators C3a and C5a are essential for liver regeneration. J Exp Med. (2003) 198:913–23. doi: 10.1084/jem.20030374 PMC2194207 12975457

[100] Panneerselvam M Bredehorst R Vogel CW. Resistance of human melanoma cells against the cytotoxic and complement-enhancing activities of doxorubicin. Cancer Res. (1987) 47:4601–07. 3621156

[101] Geller A Yan J. The role of membrane bound complement regulatory proteins in tumor development and cancer immunotherapy. Front Immunol. (2019) 10:1074. doi: 10.3389/fimmu.2019.01074 31164885 PMC6536589

[102] Liljedahl E Konradsson E Gustafsson E Jonsson KF Olofsson JK Osther K. Combined anti-C1-INH and radiotherapy against glioblastoma. BMC Cancer. (2023) 23:106. doi: 10.1186/s12885-023-10583-1 36717781 PMC9887755

[103] Arnaud L Chasset F Martin T. Immunopathogenesis of systemic lupus erythematosus: An update. Autoimmun Rev. (2024) 23:103648. doi: 10.1016/j.autrev.2024.103648 39343084

[104] Robinson WH Fiorentino D Chung L Moreland LW Deodhar M Harler MB. Cutting-edge approaches to B-cell depletion in autoimmune diseases. Front Immunol. (2024) 15:1454747. doi: 10.3389/fimmu.2024.1454747 39445025 PMC11497632

[105] de Latour RP Szer J Weitz IC Roth A Hochsmann B Panse J. Pegcetacoplan versus eculizumab in patients with paroxysmal nocturnal haemoglobinuria (PEGASUS): 48-week follow-up of a randomised, open-label, phase 3, active-comparator, controlled trial. Lancet Haematol. (2022) 9:e648–59. doi: 10.1016/S2352-3026(22)00210-1 36055332

[106] Hillmen P Szer J Weitz I Roth A Hochsmann B Panse J. Pegcetacoplan versus eculizumab in paroxysmal nocturnal hemoglobinuria. N Engl J Med. (2021) 384:1028–37. doi: 10.1056/NEJMoa2029073 33730455

[107] Lee JW Sicre DFF Wong LLL Pessoa V Gualandro S Fureder W. Ravulizumab (ALXN1210) vs eculizumab in adult patients with PNH naive to complement inhibitors: the 301 study. Blood. (2019) 133:530–39. doi: 10.1182/blood-2018-09-876136 PMC6367644 30510080

[108] Hoy SM. Pegcetacoplan: first approval. Drugs. (2021) 81:1423–30. doi: 10.1007/s40265-021-01560-8 34342834

[109] Roth A Barcellini W D’Sa S Miyakawa Y Broome CM Michel M. Sutimlimab in cold agglutinin disease. N Engl J Med. (2021) 384:1323–34. doi: 10.1056/NEJMoa2027760 33826820

[110] Bologna L Gotti E Manganini M Rambaldi A Intermesoli T Introna M. Mechanism of action of type II, glycoengineered, anti-CD20 monoclonal antibody GA101 in B-chronic lymphocytic leukemia whole blood assays in comparison with rituximab and alemtuzumab. J Immunol. (2011) 186:3762–69. doi: 10.4049/jimmunol.1000303 21296976

[111] Pawluczkowycz AW Beurskens FJ Beum PV Lindorfer MA van de Winkel JG Parren PW. Binding of submaximal C1q promotes complement-dependent cytotoxicity (CDC) of B cells opsonized with anti-CD20 mAbs ofatumumab (OFA) or rituximab (RTX): considerably higher levels of CDC are induced by OFA than by RTX. J Immunol. (2009) 183:749–58. doi: 10.4049/jimmunol.0900632 19535640

[112] Pavlasova G Mraz M. The regulation and function of CD20: an “enigma” of B-cell biology and targeted therapy. Haematologica. (2020) 105:1494–506. doi: 10.3324/haematol.2019.243543 PMC7271567 32482755

[113] de Weers M Tai YT van der Veer MS Bakker JM Vink T Jacobs DC. Daratumumab, a novel therapeutic human CD38 monoclonal antibody, induces killing of multiple myeloma and other hematological tumors. J Immunol. (2011) 186:1840–48. doi: 10.4049/jimmunol.1003032 21187443

[114] Golay J Taylor RP. The role of complement in the mechanism of action of therapeutic anti-cancer mAbs. Antibodies (Basel). (2020) 9:58. doi: 10.3390/antib9040058 33126570 PMC7709112

[115] Giorgio C Zippoli M Cocchiaro P Castelli V Varrassi G Aramini A. Emerging role of C5 complement pathway in peripheral neuropathies: current treatments and future perspectives. Biomedicines. (2021) 9:399. doi: 10.3390/biomedicines9040399 33917266 PMC8067968

